# Web-Based Alcohol Intervention: Study of Systematic Attrition of Heavy Drinkers

**DOI:** 10.2196/jmir.6780

**Published:** 2017-06-28

**Authors:** Theda Radtke, Mathias Ostergaard, Richard Cooke, Urte Scholz

**Affiliations:** ^1^ Department of Psychology Applied Social and Health Psychology University of Zurich Zurich Switzerland; ^2^ Forel Clinic Ellikon an der Thur Switzerland; ^3^ Department of Psychology Clinical Psychology and Clinical Neuropsychology University of Konstanz Konstanz Germany; ^4^ Department of Psychology School of Life and Health Sciences Aston University Birmingham United Kingdom; ^5^ University Research Priority Program Dynamics of Healthy Aging University of Zurich Zurich Switzerland

**Keywords:** attrition, dropout, alcohol drinking, intervention study, Internet, surveys and questionnaires, university student drinking, motivation

## Abstract

**Background:**

Web-based alcohol interventions are a promising way to reduce alcohol consumption because of their anonymity and the possibility of reaching a high numbers of individuals including heavy drinkers. However, Web-based interventions are often characterized by high rates of attrition. To date, very few studies have investigated whether individuals with higher alcohol consumption show higher attrition rates in Web-based alcohol interventions as compared with individuals with lower alcohol consumption.

**Objectives:**

The aim of this study was to examine the attrition rate and predictors of attrition in a Web-based intervention study on alcohol consumption.

**Methods:**

The analysis of the predictors of attrition rate was performed on data collected in a Web-based randomized control trial. Data collection took place at the University of Konstanz, Germany. A total of 898 people, which consisted of 46.8% males (420/898) and 53.2% females (478/898) with a mean age of 23.57 years (SD 5.19), initially volunteered to participate in a Web-based intervention study to reduce alcohol consumption. Out of the sample, 86.9% (781/898) were students. Participants were classified as non-completers (439/898, 48.9%) if they did not complete the Web-based intervention. Potential predictors of attrition were self-reported: alcohol consumption in the last seven days, per week, from Monday to Thursday, on weekends, excessive drinking behavior measured with the Alcohol Use Disorder Identification Test (AUDIT), and drinking motives measured by the Drinking Motive Questionnaire (DMQ-R SF).

**Results:**

Significant differences between completers and non-completers emerged regarding alcohol consumption in the last seven days (B=−.02, *P*=.05, 95% CI [0.97-1.00]), on weekends (B=−.05, *P*=.003, 95% CI [0.92-0.98]), the AUDIT (B=−.06, *P*=.007, 95% CI [0.90-0.98], and the status as a student (B=.72, *P*=.001, 95% CI [1.35-3.11]). Most importantly, non-completers had a significantly higher alcohol consumption compared with completers.

**Conclusions:**

Hazardous alcohol consumption appears to be a key factor of the dropout rate in a Web-based alcohol intervention study. Thus, it is important to develop strategies to keep participants who are at high risk in Web-based interventions.

## Introduction

### Background

According to the World Health Organization, hazardous alcohol consumption is one of the world’s leading health risks [1]. A common definition of hazardous drinking is the consumption of at least 4 (for girls or women) or 5 (for boys or men) standard glasses of alcohol (eg, 0.3 liter beer, 0.2 liter wine, or 0.04 liter spirits) on a single occasion [2]. Hazardous alcohol consumption is most common among young people [1]. For example, in Germany, young people aged 18 to 29 (1790/7649, 23.40%) are most likely to engage in hazardous drinking compared with other age groups [3]. Long-term consequences of hazardous consumption include increased risk of developing diseases such as breast cancer, coronary heart disease, and liver cirrhosis [1]. Short-term consequences of hazardous consumption include poor educational attainment and involvement in other health risk behaviors such as riding with a driver who had been drinking, using illicit drugs, and unplanned and unsafe sexual behavior [4,5]. Against the background of such health consequences, the question of why young people consume alcoholic beverages arises. According to the Motivational Model of Alcohol Use [6] 2 dimensions are relevant to classify the motivation to drink alcohol: the valence (positive or negative) and the source (internal or external) of the outcomes individuals expect to achieve from drinking alcohol. On the basis of these 2 dimensions, 4 classes of drinking motives are generated: social motives (eg, drinking to enjoy social gatherings), enhancement motives (eg, drinking to have fun), coping motives (eg, drinking to handle worries or personal problems), and conformity motives (eg, drinking to be part of the group). Among hazardous drinkers, social motives seem to be more important relative to the other motives [7].

Due to the high health risks of alcohol consumption and the high consumption among young individuals, alcohol intervention programs in young people are of key importance. Due to the increased use of the Internet by adolescents and young adults, digital media may be an effective strategy for reaching out to young people [8,9]. Furthermore, online delivery offers a cost-effective and an easy way to deliver alcohol intervention to a large number of people [10]. Web-based interventions can be as effective as traditional face-to-face interventions at reducing alcohol consumption [11,12] with the advantages of higher availability, greater convenience, and greater anonymity [13,14] when compared with the more traditional modes of delivery.

A serious concern with Web-based delivery of interventions is the potentially high rates of attrition. According to Postel, de Haan, ter Huurne, van der Palen, Becker, and Jong [15] attrition rates of Web-based alcohol interventions can be up to 92% whereas face-to-face interventions show premature termination rates of around 50% [16].

Many different reasons for attrition in Web-based intervention studies have been previously reported. Technical reasons include visual appeal, the complexity of the visual presentation, or the number of questions [17]. Other reasons include low material incentives [18,19], asking for personal information [20], non-familiarity with the Internet [15,21-23], or low interest and motivation to take part [24]. A further aspect comprises the access to guidance support (eg, via messages) instead of a self-help Web-based intervention. As shown, attrition rates are higher in Web-based intervention studies without guidance from a professional compared with Web-based studies with a counselor [25,26]. Regarding socio-demographic characteristics, evidences exist that men are more likely to be non-completers than women (eg, [15,27]) and that attrition rates are lower among participants with a lower or middle educational level compared with highly educated participants [28].

A key factor that may cause attrition is engaging in the behaviour that is the focus of the intervention [29,30]. For example, Bewick, Trusler, Mulhern, Barkham, and Hill [31] reported that among participants in their online alcohol intervention, those who completed the study reported lower alcohol consumption at baseline compared with participants who did not complete the study. Similar patterns were found in other studies (eg, [32,33]). These results support our proposition that heavier drinkers are less likely to remain in alcohol interventions [29].

Attrition threatens the internal and external validity of any intervention [34]. A key consequence of a high attrition rate among individuals at high risk is that they are unlikely to benefit from the intervention. A further key consequence of retaining people at lower risk is that intervention results, and interpretation thereof, are likely to be highly biased [35].

### The Study

Few studies to date have provided detailed analyses of attrition rates and possible reasons [36,37], and to the best of our knowledge, there are only a few studies investigating systematic attrition in Web-based alcohol interventions (eg, [15,38]) despite a high need [39]. In line with previous research [29-32] we expect that non-completers will report consuming more alcohol than completers. Relatedly, as it has been shown that hazardous drinkers report drinking for social motives more than other motives, we also expect that non-completers will be more likely to report drinking for social motives versus completers. Finally, in previous alcohol intervention studies [15,28], men were more likely to dropout than women. The current study tests the following hypotheses (H):

1. Non-completers will have a higher level of alcohol consumption compared with completers.
2. Non-completers will report higher social motives compared with completers.
3. Non-completers will be more likely to be men.

## Methods

### Participants

**Figure 1:**
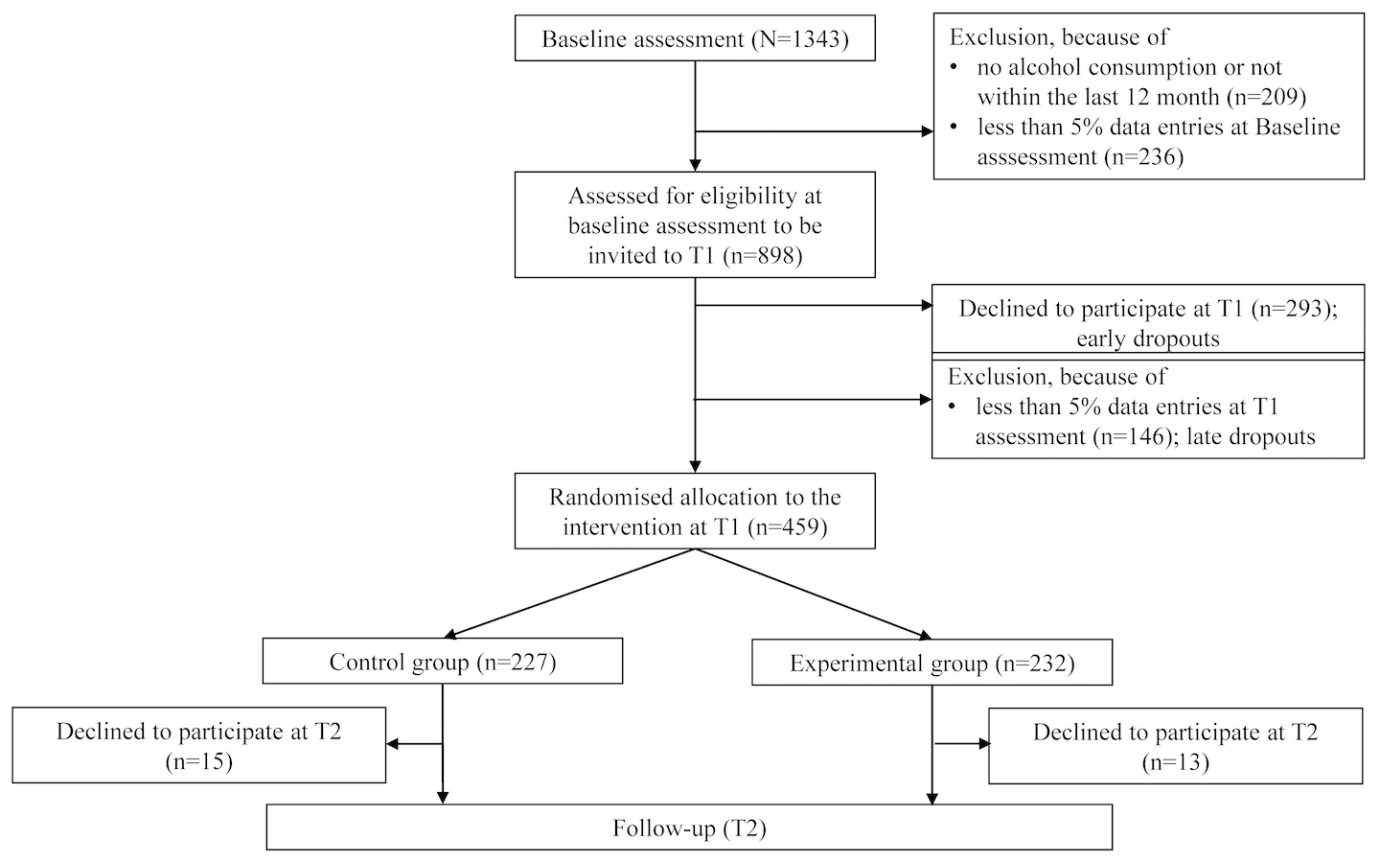
Study flow chart. T1=Time point 1, one week after baseline. T2=Follow-up, one week after T1.

Participants were recruited in Germany via the University of Konstanz student email list, flyers, posters on campus notice boards, and via social media networks (eg, Facebook). The study was advertised as a study about individual alcohol consumption habits to recruit participants who consume alcoholic beverages. The study took place during an exam free period of the semester (May and June 2012). Overall, N=1343 people clicked on the link to the online questionnaire (see Figure 1). Of those, n=209 had to be excluded because they reported that they had not consumed any alcohol in the last 12 months. In addition, n=236 were excluded as they completed less than 5% (3/53) of the questionnaire. Although one could argue that this is also a form of attrition, it was not possible to include these participants in the analyses due to too many missing values to analyze the differences between completers and non-completers. Thus, the baseline sample consisted of n=898 participants out of which 46.8% (420/898) were male and 53.2% (478/898) were female with a mean age of 23.57 years (SD 5.19). Out of the total sample, 86.9% (781/989) were students whereas the others were employees or freelancers, PhD students, school children, or interns. Women and men reported high alcohol consumption rates per week (women: mean=5.74, SD 5.01; men: mean=12.33, SD 10.93) and high alcohol consumption rates within the last seven days (women: mean=7.92, SD 7.81; men: mean=16.28, SD 15.28); this indicated hazardous alcohol consumption according to the German national drinking guidelines [2].

As an incentive to encourage participation, 10 Amazon gift cards worth 20 Euros were raffled among participants who completed all three points of measurement. Students received no credits for taking part in the study. All individuals participated voluntarily, gave informed consent, and were treated in accordance with the ethical standards of the Declaration of Helsinki [40]. This implied that the confidentiality and privacy of all participants was assured all the time. Only the main investigator had access to personal information like the e-mail address. Personal data were stored separately from data files to ensure a strictly anonymous data analysis. Furthermore, it was not possible for the participants to fill in the questionnaire twice. However, participants were able to interrupt filling out the questionnaire and return at a later time point to complete the questionnaire. All participants who did not answer the questionnaires got a reminder by email after two days.

### Design

This study was designed as a Web-based self-affirmation alcohol intervention study [41] to test possible moderators and mediators that may underlie the effects of self-affirmation on health behavior. The study design is only described briefly because the present analyses focus on the systematic dropout of participants from the intervention, rather than the intervention itself (for further information see [42]). The study consisted of 3 measurement points. The first (baseline) point of measurement took place in May and June 2012. Only participants who consumed alcoholic beverages were included in the study. After one week, participants were invited by email to take part in the study (T1) and randomly assigned to the experimental or the control group of the self-affirmation intervention (adopted from [41,43]). Participants in the experimental condition had to choose one of their most important values from a list of several values (eg, reliability) and to write down why this value is important to them. In the control condition, participants were instructed to write about their least important value from the listed values and why it might be important to another person. After this intervention task, all participants had to read health messages about alcohol consumption. This information included a description of short-term and long-term health risks of heavy alcohol consumption (eg, cancer), a definition of heavy drinking, and the recommended intake of alcoholic beverages. Afterwards, participants had to fill in several measures. The final follow-up (T2) took place one week later. Again, participants were invited by email to take part in the study.

### Measures

The following instruments were included in the online questionnaire at the baseline measurement one week prior to the intervention.

Alcohol Consumption (adapted from [41]). Alcohol consumption was assessed with 4 items. Before answering the items, participants were provided with a brief definition and examples of standard glasses of different alcoholic beverages in line with the German drinking guidelines [44]. An example is: “One German standard drink equals 10g to 12g of pure alcohol, which equates to a small beer (250 ml), or a small glass of wine (100 ml).” The items concerning alcohol-consumption were ‘How many standard drinks do you consume per week?’ (per week), ‘Referring to the last 7 days, how many standard drinks did you consume?’ (last seven days), ‘How many standard drinks do you consume from Monday to Thursday?’ (Monday to Thursday), and ‘How many standard drinks do you consume from Friday to Sunday?’ (on weekends).

The Alcohol Use Disorders Identification Test (AUDIT, [45]) measures excessive drinking behavior. The 10-item questionnaire screens for harmful alcohol use. An item example is, ‘How often do you have six or more drinks on one occasion?’ A sum score of eight or above refers to hazardous alcohol consumption, a score between 16 and 19 refers to harmful alcohol consumption, and a score higher than 20 indicates a possible alcohol dependence. Cronbach alpha of the scale was equal to .83.

The Drinking Motive Questionnaire Revised Short Form (DMQ-R SF, [46]) measures 4 different motives to drink alcohol. The social (Cronbach alpha =.84), enhancement (Cronbach alpha=.76), coping (Cronbach alpha=.86), and conformity (Cronbach alpha=.79) motive dimensions were assessed with three items each. The 5-point Likert-scale ranges from 1 = (almost) never to 5 = (almost) always. The stem for each item was ‘I have drunk alcohol in the past 12 months’ followed by different item options, for example, ‘...in order to be liked’. Cronbach alpha for the overall scale was equal to .83.

### Data Analysis

All analyses were run using SPSS version 20 (IBM, Somers, NY, USA) to test the hypotheses. In a first step, *t* tests and Chi-square tests (threshold for statistical significance *P*<.05) were conducted to reveal group differences concerning alcohol consumption, excessive drinking behavior (AUDIT), drinking motives (DMQ-R SF), and socio-demographics like gender, age, and student status. After the detection of differences between completers and non-completers, the relationship between dropout classification and possible predictors was tested with logistic regressions in a second step. Logistic regression analyses were performed with completion of the Web-based self-affirmation intervention as the dependent variable. Predictor variables showing a significant association with dropout (based on correlations) were entered into the model, whereas non-significant variables were removed. In addition, it was decided to include only one variable regarding alcohol consumption in the model to avoid multicollinearity as the variables correlated to a high degree. Thus, 4 models were calculated. The goodness of fit of the models was evaluated by the Hosmer-Lemeshow test, which needs to be non-significant.

## Results

Overall, the baseline sample consisted of n=898 participants. Of these, 293 participants did not participate at T1 (early dropouts). A further n=146 participants who began completing the online questionnaire at T1 dropped out (ie, completed less than 5% data of the questionnaire) before being allocated to one of the experimental conditions (late dropouts; see Figure 1).

Overall, no significant differences were found between the early and late dropouts regarding most of the measures. The only two significant differences were found regarding the AUDIT score and the amount of alcohol consumption on weekends. Early dropouts reported significantly higher AUDIT scores compared with the late dropouts (mean=9.60 vs mean=8.47; *t*_317.76_=2.19, *P*=.03, *d*=0.21, 95% CI [0.12-2.13]) as well as higher alcohol consumption on weekends (mean=8.37 vs mean=6.90; *t*_335.46_=2.16, *P*=.03, *d*=0.21, 95% CI [0.13-2.82]). On the basis of these few differences and the fact that the results of the logistic regression revealed an almost identical pattern of results when running the analyses separately for early and late dropouts, it was decided not to further distinguish between both groups in the following analyses. Overall, the dropout rate from baseline to the randomized allocation to the intervention at T1 was 48.9% (439/898). At T2, a further n=28 dropped out. However, those non-completers did not differ significantly from the completers regarding all measures, except for the sub-dimension coping motives of the DMQ-R SF (mean=2.08 vs mean=1.59; *t*_457_=−3.44, *P*=.001, *d*=0.67, 95% CI [−0.77 to −0.21]).

Tables 1 and 2 present the descriptive statistics for completers (n=459) and non-completers (n=439). Significant differences between completers and non-completers were found regarding alcohol consumption, AUDIT score, coping, enhancement and social drinking motives, gender, and student status. Non-completers showed a significantly higher alcohol consumption per week, from Monday to Thursday, on weekends and in the last seven days. In addition, non-completers reported significantly higher AUDIT scores and higher mean values regarding social, enhancement, and coping motives for drinking alcohol compared with completers. Furthermore, women and students were more likely to be completers compared with men and non-students. No significant differences between completers and non-completers were found for age, or the conformity motive.

**Table 1:** Sample characteristics (mean, SD) and differences concerning systematic attrition.

Sample characteristics		Attrition							
		Completers N=459		Non-completers N=439		*All*	*All*		
		Mean	SD	Mean	SD	T	*P*	95% CI	Cohen d
**Alcohol consumption (standard drinks)**									
	per week (range 0-70)	7.20	6.85	10.60	10.50	−5.71	<.001	−4.56 to −2.23	.39
	Monday to Thursday (range 0-40)	2.60	3.58	3.96	5.37	−4.45	<.001	−1.96 to −0.76	.30
	Weekends (range 0-45)	5.18	4.76	7.88	7.15	−6.63	<.001	−3.50 to −1.90	.45	
	last seven days (range 0-95)	9.65	9.54	14.17	14.83	−5.41	<.001	−6.16 to −2.88	.36
AUDIT^a^ (range 0-27)		7.19	4.20	9.22	5.27	−6.35	<.001	−2.65 to −1.40	.43
**Drinking motives (range 1-5)**									
	social motives	3.15	.92	3.30	.98	−2.33	.02	−0.27 to −0.02	.13	
	enhancement motives	2.88	.92	3.08	.98	−3.22	.001	−0.32 to −0.08	.21
	coping motives	1.62	.74	1.78	.85	−2.98	.003	−0.26 to −0.05	.20
	conformity motives	1.33	.57	1.35	.61	−.55	.58	−0.10 to 0.06	-
Age (range 16-67)		23.51	5.17	23.63	5.22	−.33	.74	−0.80 to 0.57	-

^a^AUDIT: Alcohol Use Disorders Identification Test.

**Table 2:** Distribution of sample characteristics and differences concerning systematic attrition.

Sample characteristics	Attrition						
	Completers N=459		Non-completers N=439			
	N	%	N	%	χ^2^	*P*
Female	269	58.6	208	47.6	10.90	.001
Student	416	90.6	360	82.0	12.61	<.001

**Table 3:** Intercorrelations between variables separated by completers (upper half) and non-completers (lower half).

	Completers											
Non-Completers	1^a^	2^b^	3^c^	4^d^	5^e^	6^f^	7^g^	8^h^	9^i^	10^j^	11^k^	12^l^
1^a^	-	.82 <.001	.86 <.001	.74 <.001	.71 <.001	.23 <.001	.28 <.001	.21 <.001	.08 .071	−.03 .57	.34 <.001	.02 .75
2^b^	.83 <.001	-	.57 <.001	.59 <.001	.57 <.001	.13 .004	.19 <.001	.21 <.001	.06 .19	.00 .99	.26 <.001	.04 .37
3^c^	.87 <.001	.63 <.001	-	.71 <.001	.68 <.001	.24 <.001	.28 <.001	.16 .001	.04 .35	−.06 .24	.29 <.001	−.01 .91
4^d^	.80 <.001	.66 <.001	.73 <.001	-	.61 <.001	.26 <.001	.26 <.001	.16 <.001	.05 .31	−.03 .51	.29 <.001	.01 .82
5^e^	.61 <.001	.47 <.001	.61 <.001	.56 <.001	-	.43 <.001	.39 <.001	.36 <.001	.09 .06	−.12 .009	.33 <.001	.04 .35
6^f^	.25 <.001	.18 <.001	.29 <.001	.21 <.001	.48 <.001	-	.57 <.001	.16 .001	.25 <.001	−.25 <.001	.18 <.001	.13 .006
7^g^	.31 <.001	.24 <.001	.36 <.001	.26 <.001	.47 <.001	.63 <.001	-	.23 <.001	.21 <.001	−.11 .02	.12 .01	.07 .16
8^h^	.14 .004	.11 .02	.17 <.001	.14 .003	.41 <.001	.27 <.001	.31 <.001	-	.13 .006	−.01 .94	−.07 .13	−.03 .49
9^i^	.03 52	.02 .70	.03 .50	.02 .70	.26 <.001	.36 <.001	.28 <.001	.20 <.001	-	−.09 .07	.07 .17	.07 .12
10^j^	.02 .65	.05 .28	−.02 .69	.01 .83	−.09 .06	−.29 <.001	−.11 .02	.01 .84	−.02 .69	-	−.05 .34	−.51 <.001
11^k^	.38 <.001	.25 <.001	.32 <.001	.35 <.001	.34 <.001	.13 .008	.08 .10	−.06 .20	.08 .09	.03 .58	-	−.05 .30
12^l^	−.02 .74	.05 .26	−.06 .19	−.01 .97	.02 .69	.16 .001	.03 .56	−.04 .44	.01 .78	−.46 <.001	−.10 .04	-

^a^Alcohol consumption per week.

^b^Alcohol consumption Monday-Tuesday.

^c^Alcohol consumption weekends.

^d^Alcohol consumption last seven days.

^e^Alcohol Use Disorders Identification Test (AUDIT).

^f^Social drinking motives.

^g^Enhancement drinking motives.

^h^Coping drinking motives.

^i^Conformity drinking motives.

^j^Age.

^k^Gender; 1=male, 0=female.

^l^Student; 1=student, 0=non-student.

In Table 3 the intercorrelations separated by completers and non-completers are presented. As evident, the intercorrelations among the great majority of the study variables are similar across completers and non-completers. Overall, completion of the study was significantly negatively related with all 4 alcohol consumption measures (*r*=−.15 to −.22, *P*<.001), the AUDIT score (*r* =−.21, *P*<.001), all drinking motives except for conformity drinking motives (*r* =−.08 to *r* =−.11, *P*=.001 to .02), gender (*r* =−.11, *P*=.001), and status as a student (*r* =−.12, *P*<.001).

Four multivariate logistic regression analyses were performed to predict the completion of Web-based alcohol intervention using the variables outlined above. Results across the 4 analyses revealed that alcohol consumption on weekends and in the last seven days, the AUDIT score, and being a student predicted completion of the study (see Table 4). The higher the alcohol consumption on weekends and in the last seven days as well as the AUDIT score, the more likely an individual was to be a non-completer. Students were more likely to complete the study than non-students. All other variables included in the model did not significantly predict study completion. Overall, the regression models showed sufficient goodness of fit.

**Table 4:** Logistic regression of completion^a^ of the Web-based self-affirmation intervention.

Scale			B	SE	*P*	Odds ratio	95% CI for odds ratio
**Constant Model 1**			.30	.33	.36	1.35	
	**Alcohol consumption (standard drinks)**						
		per week	−.02	.01	.08	.98	0.96-1.00
	AUDIT^b^		−.06	.02	.02	.95	0.90-0.99
	**Drinking motives**						
		social motives	.03	.10	.75	1.03	0.85-1.25
		enhancement motives	−.06	.09	.55	.95	0.79-1.14
		coping motives	−.07	.10	.46	.93	0.77-1.13
	Gender^c^		−.11	.15	.48	.90	0.66-1.21
	Student^d^		.72	.21	.001	2.04	1.35-3.11
	R^2^=.06 (Cox & Snell), .08 (Nagelkerke). Model Model χ^2^_8_=5.05, *P*=.75. Correct classification 60.7%.					
**Constant Model 2**			.28	.33	.38	1.33	
	**Alcohol consumption (standard drinks)**						
		Monday to Thursday	−.03	.02	.18	.97	0.94-1.01
	AUDIT^b^		−.07	.02	.002	.94	0.90-0.98
	**Drinking motives**						
		social motives	.04	.10	.69	1.04	0.86-1.26
		enhancement motives	−.07	.09	.49	.94	0.78-1.13	
		coping motives	−.06	.10	.52	.94	0.78-1.14
	Gender^c^		−.14	.15	.36	.87	0.65-1.17
	Student^d^		.73	.21	.001	2.08	1.34-3.15
	R^2^=.06 (Cox & Snell), .08 (Nagelkerke). Model χ^2^_8_=4.36, *P*=.82. Correct classification 51.3%.					
**Constant Model 3**			.33	.33	.31	1.39	
	**Alcohol consumption (standard drinks)**						
		Weekends	−.05	.02	.003	.95	0.92-0.98
	AUDIT^b^		−.04	.02	.08	.96	0.92-1.0
	Drinking motives						
		social motives	.03	.10	.75	1.03	0.85-1.25
		enhancement motives	−.04	.10	.67	.96	0.80-1.16
		coping motives	−.08	.10	.38	.92	0.76-1.11
	Gender^c^		−.11	.15	.46	.89	0.66-1.21
	Student^d^		.68	.22	.002	1.97	1.29-2.99
	R^2^=.07 (Cox & Snell), .09 (Nagelkerke). Model χ^2^_8_=8.69, *P*=.37. Correct classification 61.0%.					
**Constant Model 4**			.30	.33	.36	1.35	
	**Alcohol consumption (standard drinks)**						
		last seven days	−.02	.01	.05	.99	0.97-1.00
	AUDIT^b^		−.06	.02	.007	.94	0.90-0.98
	**Drinking motives**						
		social motives	.04	.10	.68	1.04	0.86-1.26
		enhancement motives	−.06	.09	.52	.94	0.78-1.13
		coping motives	−.07	.10	.47	.93	0.77-1.13
	Gender^c^		−.11	.15	.46	.89	0.66-1.21
	Student^d^		.72	.21	.001	2.05	1.35-3.11
	R^2^=.06 (Cox & Snell), .08 (Nagelkerke). Model χ^2^_8_=4.65, *P*=.80. Correct classification 60.3%.					

^a^Completion: 0=non-completers, 1=completers.

^b^AUDIT: Alcohol Use Disorders Identification Test.

^c^Gender: 1=male, 0=female.

^d^Student: 1=student, 0=non-student.

## Discussion

### Principal Findings

This study found that individuals who reported higher alcohol consumption on weekends and in the last seven days were less likely to complete a Web-based alcohol intervention over and above other predictors, providing support for Hypothesis 1. In addition, higher likelihood of problematic alcohol consumption as indicated by higher AUDIT scores positively predicted the dropout rate over and above other predictors. In line with our Hypotheses 2 and 3, non-completers reported higher levels of social drinking motives compared with completers, and men were more likely to dropout compared with women. However, social drinking motives and gender did not predict completion of the study over and above other predictors, so Hypotheses 2 and 3 have to be rejected. However, we found that students were more likely to complete a Web-based alcohol intervention than non-students.

As assumed in Hypothesis 1, non-completers showed higher levels of alcohol consumption compared with completers. There are several explanations for the higher attrition rate among individuals with higher alcohol consumption and higher AUDIT scores. Participants with higher alcohol consumption and AUDIT scores may have responded with defensive reactivity [41] when confronted with the definition of a standard drink and further information about acceptable and excessive alcohol consumption, which could have led to dissatisfaction with the study in general or the intervention in particular and consequently to a dropout (see also [15]). Another explanation for early termination of the study could be the experience of cognitive dissonance [47] in individuals with higher alcohol consumption and AUDIT scores when learning about the German drinking guidelines and evaluating their drinking against these guidelines. Early termination of study participation could have been one way to resolve their cognitive dissonance. In line with this, participants might also have terminated the intervention as they might have recognized that a reduction of their level of alcohol consumption is not realistic. However, all these explanations remain speculative and can account only in some parts for the finding of a higher attrition rate among individuals with a high alcohol consumption. Therefore and because only very few studies exist that examined reasons for dropout [48], future research should examine in more detail why individuals at risk (especially regarding alcohol consumption) have higher attrition rates.

However, some differences emerged regarding the different measures of alcohol consumption included in this study. Despite significant mean differences between completers and non-completers concerning alcohol consumption per week as well as the alcohol consumption from Monday to Thursday, these two alcohol measures did not significantly contribute to the prediction of the dropout rate in the regression analyses in comparison to other predictors. This provides an important information on the characteristics of a group that might be especially hard to reach: this study’s results indicate that it might be especially important to address interventions in young adults engaging in excessive weekend drinking [49]. These results also emphasize the importance of assessing more than a single indicator of alcohol consumption as was done in this study. This is of high importance especially for heavy alcohol drinkers or alcohol abusers. Several explanations have been offered why those individuals tend to underreport their alcohol consumption. Other than forgetting, the way how alcohol consumption was measured might also lead to biased self-reports [50]. To resolve such problems with self-reports, Sobell and Sobell [50] recommend including a minimum set of essential items, such as, usual quantity of drinking. In addition, future studies might also want to complement self-reported alcohol consumption with objective measures such as blood alcohol concentration, the enzymes gamma glutamyl transpeptidase (gamma-GTP), glutamic-oxaloacetic transaminase (GOT), or glutamic-pyruvic transaminase (GPT) [50,51]. However, objective measurements are not suitable for all alcohol-related studies like Web-based surveys or interventions like in this study. The reason is that an objective measurement of alcohol consumption requires a face-to-face contact with participants. In addition, there are also shortcomings of objective measurements. For example, the measurement of gamma-GTP does not guarantee to be a sole indicator of excessive alcohol consumption as an increased serum gamma-GTP activity is also associated with overweight [51]. The same problem of the causal relationship applies to the assessment of the GOT and GPT levels. These objective measures provide information about the severity of the liver disease, which might be a consequence of excessive alcohol consumption [52].

Contrary to Hypothesis 2 drinking motives were not significant predictors of study completion in contrast to other predictors like alcohol consumption as revealed by logistic regression analyses. However, significant mean differences between completers and non-completers emerged regarding drinking motives. As expected, non-completers reported higher social drinking motives compared with completers. In addition, coping and enhancement drinking motives were higher among non-completers [7,53], whereas there was no difference regarding conformity motives. As non-completers in this study are characterised as individuals with high alcohol consumption, this result is in line with research showing that conformity motives are generally not related to alcohol consumption (eg, [54]). Concerning Hypothesis 3, it was found that more men than women dropped out in the current study. This is in line with the result that men reported higher alcohol consumption compared with women as also found in other studies [15,28]. However, as gender did not emerge as a significant predictor in the logistic regression analysis, it appears that alcohol consumption is a more important factor for attrition than gender.

An additional predictor of completing the study was student status. Students were more likely to complete the study as compared with non-students. This result is in contrast to previous research, which did not find a difference regarding the level of education and attrition in Web-based studies [15,55]. However, previous studies also reported that individuals with higher education and higher socioeconomic status are more likely to take part in psychological surveys and interventions (eg, [56,57]). Thus, further research is recommended to focus on the socioeconomic status and the level of education as a predictor of completion of a Web-based intervention; as alcohol-related harms are more likely in individuals with lower educational attainment and or lower socioeconomic status this is a key issue to resolve. Furthermore, it might also be the case that students are more familiar with the participation in Web-based studies. The reason for that might be that numerous studies, lecture evaluations, or surveys via Internet take place at the University of Konstanz. This might enhance the probability that students have lower attrition rates in contrast to non-students. In addition, it might also be the case, that students are more willing to spend time filling out further questionnaires compared with non-students as non-students might be less flexible due to a more stringent working schedule. Furthermore, it might be possible that amazon vouchers that were raffled among participants were more attractive for students due to a tight budget compared with non-students.

### Limitations

This study is not without limitations. First, all the information was derived from self-reports, which might cause a bias due to social desirability and memory effects like forgetting [50,58]. Therefore, future research is recommended to use objective measurements (eg, breathalyzer for estimating blood alcohol concentration, see discussion above) although self-reported alcohol consumption has been shown to be reliable and valid [50,59]. However, the validity and reliability depends upon the accurate measure of alcohol consumption. Therefore, standardized alcohol consumption measures should be used. In this study one of the 4 alcohol measures (AUDIT) was a standardized one. Nonetheless, the other measures included in this study followed Sobell and Sobell`s recommendations how to best measure alcohol consumption [50].

A second limitation of the study is that the sample consisted mainly of students (781/898, 86.4%). As outlined above, a greater heterogeneity in socioeconomic status would be desirable to learn more about the characteristics of people at most risk for hazardous alcohol consumption. However, as alcohol consumption in students is a problem per se [7,31,32,60], this study is nonetheless able to make a significant contribution to the identification of risk factors for attrition of alcohol-intervention studies.

### Conclusions

Reducing attrition is essential for any intervention. Usually, interventions are designed to reach people who are at the most risk. Unfortunately, as shown by this study, individuals with higher alcohol consumption were more likely to dropout before or at the beginning of the intervention. Thus, it is important to develop and test different strategies to keep participants who are at high risk in Web-based interventions. Only after taking this into account can the effectiveness of Web-based interventions for individuals at high risk be evaluated correctly.

## Abbreviations

AUDIT: Alcohol Use Disorder Identification Test
DMQ-R SF: Drinking Motive Questionnaire Revised Short Form
T1: Time point 1, one week after baseline
T2: Follow-up, one week after T1
